# Who Tweets? Deriving the Demographic Characteristics of Age, Occupation and Social Class from Twitter User Meta-Data

**DOI:** 10.1371/journal.pone.0115545

**Published:** 2015-03-02

**Authors:** Luke Sloan, Jeffrey Morgan, Pete Burnap, Matthew Williams

**Affiliations:** 1 Cardiff School of Social Sciences, Cardiff University, Cardiff, United Kingdom; 2 Cardiff School of Computer Science and Informatics, Cardiff University, Cardiff, United Kingdom; University of Warwick, UNITED KINGDOM

## Abstract

This paper specifies, designs and critically evaluates two tools for the automated identification of demographic data (age, occupation and social class) from the profile descriptions of Twitter users in the United Kingdom (UK). Meta-data data routinely collected through the Collaborative Social Media Observatory (COSMOS: http://www.cosmosproject.net/) relating to UK Twitter users is matched with the occupational lookup tables between job and social class provided by the Office for National Statistics (ONS) using SOC2010. Using expert human validation, the validity and reliability of the automated matching process is critically assessed and a prospective class distribution of UK Twitter users is offered with 2011 Census baseline comparisons. The pattern matching rules for identifying age are explained and enacted following a discussion on how to minimise false positives. The age distribution of Twitter users, as identified using the tool, is presented alongside the age distribution of the UK population from the 2011 Census. The automated occupation detection tool reliably identifies certain occupational groups, such as professionals, for which job titles cannot be confused with hobbies or are used in common parlance within alternative contexts. An alternative explanation on the prevalence of hobbies is that the creative sector is overrepresented on Twitter compared to 2011 Census data. The age detection tool illustrates the youthfulness of Twitter users compared to the general UK population as of the 2011 Census according to proportions, but projections demonstrate that there is still potentially a large number of older platform users. It is possible to detect “signatures” of both occupation and age from Twitter meta-data with varying degrees of accuracy (particularly dependent on occupational groups) but further confirmatory work is needed.

## Introduction

Within the natural and social sciences the advent of ‘big data’ and all the problems associated with it are feeding a methodological and analytical revolution. Whilst 90% of the world’s data has been generated in the past 2 years [1] and the trend is apparently exponential, the key challenges of harnessing this data (known as the 5Vs: volume, veracity, velocity, variety and value) are not so easily overcome. Indeed, the term ‘big data’ itself is less than precise as it refers to a vast range of transactional and naturally occurring sources. However it would be inaccurate to claim that our ability to generate and record vast quantities of data has completely outpaced our methodological toolkit, rather it could be argued that the challenges of making sense of ‘big data’ have caused scholars to ask new questions about what can be known and develop new methods for finding answers. Notably transactional data generated during internet searches has been used to track the spread of flu in the US [2] and even to build psychological constructs of nations linked to GDP [3]. Predictive models of box office revenue for new films have been built using Wikipedia activity [4], internet search term frequency [5] and Twitter word of mouth [6]. Indeed, increasing attention is being given to the role of big data in understanding collective behaviour in the real world (see [7] for an excellent summary of the area). Social media has also been used to investigate fluctuations in the USD/EUR exchange rate [8][9] and to assign psychological profiles and demographic characteristics to Facebook users based on their vocabulary [10]. Even the digitalisation of paper books has enabled researchers to follow new lines of enquiry that were previously impossible, such as how languages change over time [11].

Conducting such studies requires a step-change in how researchers typically conduct investigations using ‘big data’ and reconsideration of what methods, approaches and research designs are appropriate for naturally occurring data (as opposed to data elicited through survey research, for example). The primary criticism of such data is that it is there to be collected and analysed before the question is asked and, because of this, the data required to answer the research question may not be available with important information such as demographic characteristics being absent.

Within 21^st^ Century social sciences there is an increasing body of research concerned with the viability of the use of naturally occurring data produced via social media for social scientific analysis and the issue of missing demographic information is a key barrier to researchers. The advent of ‘big and broad data’ provides a potential goldmine of data on public opinion, social networks and interactions, identity and cultural values which is both user generated and, for some data sources, free to access. Indeed, there are approximately 1.3 billion monthly active users of Facebook [12] who alone upload approximately 500 terabytes of data every day with just under 0.5 billion status updates [13]. For the social sciences, the sheer volume of data and its constant, flowing, locomotive nature provides an opportunity to take the ‘pulse of the world’ every second of the day rather than relying on punctiform and time-consuming terrestrial methods such as surveys.

However, social media data sources that are both abundant and easy to access, Twitter data for example, are often considered to be ‘data-light’ as there is a paucity of demographic information on individual content producers leading some academics to dismiss social media as a potentially rich source of social science data [14][15]. Yet, as Savage and Burrows argue [16], sociology (and more broadly the social sciences) needs to respond to the emergence of these new data sources and investigate the ways in which they inform us of the social world. One response to this has been the development of using ‘signatures’ in social media as proxies for real world events and individual characteristics [17][18], advocating the use of indirect and incidental information associated with sources of ‘big data’ to scope and observe digital sociological phenomena, and the argument that social media is not a surrogate for traditional terrestrial methods of data collection but an opportunity to augment them [19]. Following from this, Sloan et al. [20] have demonstrated that there are ways in which demographic information on location, gender and language can be derived from Twitter data by the use of proxies and/or associated meta-data thus repackaging social media content for social scientific analysis. This paper builds on this work conducted at the Collaborative Online Social Media ObServatory (COSMOS) [21], through proposing methods and processes for estimating two more important demographic variables: age and occupation (with associated class). We propose, enact and critically evaluate methods for identifying both demographic characteristics through the lens of ‘signature social science’ as an incremental step in increasing the utility of social media data for social scientific analysis.

We approach the estimation of each demographic characteristic individually, as the methods through which they can be extracted differ. For occupation and class we begin by discussing the link between the two using the reference tables provided by the Office for National Statistics (ONS) on Standard Occupational Classification 2010 (SOC2010) and National Statistics Socio-Economic Classification (NS-SEC). This is followed by a discussion of access to Twitter data and the associated meta-data fields from which we derive occupational information. Close attention is paid to the reliability of the data and we discuss some of the problems we anticipated, particularly around type 1 errors and the manner in which people may chose to present themselves in their Twitter profile. We then specify the rules used to build the occupation detection algorithm and how special cases are handled and move on to an evaluation of the initial results of the human validation of a random test sample. Following this we present the NS-SEC profile of Twitter users, making comparisons between the results of the automated tool, cases in which human expert coders agreed, all cases in our database to which an occupation could be assigned and the general UK population (using 2011 Census data).

For age we briefly discuss previous attempts to identify the age of social media users on other platforms based on vocabulary and word frequency and we explore why these methods cannot simply be ported between social media platforms, drawing on the idiosyncrasies of Twitter in particular. The rules used to build a detection tool are discussed and we present the age profile of Twitter users alongside the age profile of the 2011 UK Census population, commenting on the different distributions and issues around reliability and accuracy.

To conclude, future avenues of research are suggested including ideas for how accurate baseline data could be collected to verify the effectiveness and reliability of proxy demographic variables via social surveys. In particular we advocate the need to cross-reference alternative data sources with social media to explore the potential discrepancies between who and what people are and how they choose to represent themselves online. Attention is also given to the ethical implications for social scientists handling social media data.

## Occupation and Class

### Data

The COSMOS class engine is designed to identify the socio-economic group (NS-SEC) of a tweeter through the identification of occupation. NS-SEC measures employment relations and conditions of occupations [22][23][24] which “are central to showing the structure of socio-economic positions in modern societies and helping to explain variations in social behaviour and other social phenomena” [25]. It is the standard measure of class used by government and researchers to map changing trends over time, quality of work and inter-generational social mobility and has influenced cross-national measures of class to allow international comparative analysis [24]. There are 8 NS-SEC analytic classes (see Table 1[25]), with group one split into two sub categories that distinguishes between higher managerial and higher professional occupations: To locate an individual within an NS-SEC group it is necessary to take into account both their occupation *and* other details of their employment status such as whether they are employed or self-employed and the size of the organisation in which they work. However, the use of occupation alone as a proxy for NS-SEC group is common in social data and it is routine to use look-up tables provided by the Office for National Statistics to link an occupation to a standard occupational classification code (SOC2010) and then from SOC2010 to simplified NS-SEC [25]. Such matching is regularly carried out by organisations such as UCAS who locate a HE student applicant in an NS-SEC group based on the occupation of the main earner in a household using the following question:

"If you are in full-time education, please state the occupation of the highest-earning family member of the household in which you live. If he or she is retired or unemployed, give their most recent occupation. If you are not in full-time education, please state just your own occupation."

(HESA 2014) [26]

**Table 1:** NS-SEC Analytic Categories (Source: ONS 2014).

***Group*:**		***Description*:**
*1*		*Higher managerial*, *administrative and professional occupations*
	*1.1*	*Large employers and higher managerial and administrative occupations*
	*1.2*	*Higher professional occupations*
*2*		*Lower managerial*, *administrative and professional occupations*
*3*		*Intermediate occupations*
*4*		*Small employers and own account workers*
*5*		*Lower supervisory and technical occupations*
*6*		*Semi-routine occupations*
*7*		*Routine occupations*
*8*		*Never worked and long-term unemployed*

*Note that ‘students’*, *‘occupations not stated’ and ‘not classifiable’ are treated as ‘not classified’ and are not included in this table*.

This simplification is necessary when applying a ‘signature science’ lens to naturally occurring data through which limited information about an individual's’ workplace environment is available.

Having identified occupation as the starting point for assigning NS-SEC groups, we turn to the problem of extracting occupational information from Twitter data. Through querying the Twitter API via the 1% ‘spritzer’ feed [27] we can extract various meta-data fields associated with each individual user. One of these fields is a limited open text description in which the user can choose to write whatever they like. Sometimes this is left blank but normally this is a statement about attitudes or beliefs, hobbies and even information relating to employment and occupations. The critical point to make is that, even if only 1% of all Twitter users in the UK include information about their occupation in this field, this still leaves us with the employment information for around 150,000 users. Such is the scale of social media participation that even a tiny kernel of ‘useful’ data that accounts for a very small proportion of total users can still amount to a sample bigger than some of the UK’s largest sample surveys—this is the latent power of naturally occurring big and broad data [27].

By using the SOC2010 look-up table provided by the ONS we cross-referenced all text from this open field against a sample of tweets collected by COSMOS as part of our standard archiving of the gratis 1% feed focusing on UK tweets and we then visually inspected the results in anticipation of inaccuracies and errors arising from such a simple pattern matching ruleset. This first iteration was intentionally explorative and highlighted a series of problems that needed to be overcome—the first of which was the presence of multiple occupations. It became clear that many Twitter users use the open text field to list the many roles that they take on in their daily lives and we have no method of identifying the ‘primary’ or most salient role as perceived by that individual, for example:

“Lecturer in social science plus amateur horror writer and keen fair-weather gardener”

Although this example is fabricated, it is typical of the data and contains 3 potential occupations which span the NS-SEC analytic groups: ‘Lecturer’ (SOC2010 2311, NS-SEC 1.2), ‘Writer’ (SOC2010 2471, NS-SEC 2) and ‘gardener’ (SOC2010 5113, NS-SEC 4). The problem of identification is further complicated by the fact that ‘Gardener’ could also refer to at least three SOC2010 codes which all fall into different NS-SEC classifications: 5112 horticultural trades (NS-SEC 6), 5114 groundsmen and greenkeepers (NS-SEC 5), 5113 gardeners and landscape gardeners (NS-SEC 4). In response to this we make the decision that the most salient occupation of an individual (i.e. the role they most identify with) is the occupation they mention first, thus we programme our pattern matching algorithm to look for the first instance of a SOC2010 occupation and to move on to the next case once this has been found (or if nothing matching is found at all). We also match the maximum number of words possible, so a landscape gardener would be matched as so rather than simply a gardener.

An additional concern specifically for occupation identification is that Twitter users often use their profile description to list their hobbies rather than or in addition to the occupations that they are employed in. In the example given above it is reasonable to assume that the tweeter is not a lecturer in social science as a hobby, but that horror writing and gardening are leisure activities that they enjoy. When the occupation is listed first then this is not a concern as the pattern matching algorithm will identify the correct occupational role, but there is no reason to assume that this is the norm, particularly if social media users primarily identify with what is most important to them which may not be their employment. Indeed, one of the reasons for human validation of the data (discussed below) is to test the efficacy of taking the first occupation listed through human identification and attempting to learn from type 1 errors.

Our fourth problem is one of false positives. The SOC2010 list of occupations has 28,019 entries some of which appear obscure due to their historical nature; however by far the most significant problem when identifying occupation from profiles is the overlap in terminology. As an example, let us consider a slightly modified version of the fabricated description used previously:

*“Card player*, *lecturer in social science and keen fair-weather gardener”*

The first SOC2010 occupational term that arises in this description is ‘player’ which can refer to a ‘team player’ in retail, a ‘player’ of musical instruments or a ‘player’ of sports. Of course, the tweeter falls into none of the three possible variants of ‘player’ as they are identifying with a leisure activity (which, if they are any good at it, may well also provide their primary income). We report on the prevalence of obscure occupational roles in the results section.

Apart from misclassification, there is always the possibility that users are simply lying about their occupation (and, indeed, their age) and we acknowledge that Internet users engage in identity-play, and that this phenomenon can result in incorrect classifications of ‘offline’ categories such as occupation. The notion of a ‘virtual identity’ in relation to the Internet was first popularized by Turkle [28] who struggled with issues of identity of online respondents and the authenticity of their responses at an early point in the Internet’s history. In these early years of limited adoption, users’ primary engagement was via game-like environments (e.g. Multi-User Domains) and hobbyist Newsgroups and Bulletin Boards. Given the nature of these environments users would often portray alternative online personas [29][30]. However, research also indicated that identity-play and the adoption of alternative personas was often short-lived, with ‘real’ users’ identities becoming dominant in prolonged interactions [31]. The exponential uptake of the Internet, beyond this particular group of early adopters, was accompanied with a shift in the presentation of self online resulting in a reduction in online identity-play [32]. This shift was partly due to the rise of Web2.0 and the democratization of multi-media content production. The ability to upload images and video, as well as text encouraged users to portray their ‘offline’ lives above possible online fantasy alternatives [33]. This is particularly the case with users who use social media for professional purposes, in whole or in part [34]. While online identity-play remains a feature of the Internet, we argue that those including occupation information in their Twitter meta-data are less likely to engage in the activity given the likely (semi)professional nature of their accounts. That said, we must accept that some users have provided false occupational information and acknowledge this as a shortcoming of this form of data, although it is important to note that even with traditional offline methods, social researchers have to routinely trust respondents’ answers to demographic questions without any way of verifying authenticity.

### Occupation Detection Algorithm

To extract the occupation from the description field of a Twitter user, we start by cleaning the text to remove line breaks and non-alphabetic characters. This cleaning process follows a similar process that we described in our previous work [20] to mitigate the creative use of spacing and punctuation in Twitter profile descriptions. Next, we extract the occupation by searching the description field for occupations that occur in the SOC2010 list. Occupations can have more than one word (such as ‘landscape gardener’ and ‘electrical engineer’). In many cases, the words in a multiple-word occupation also match occupations with fewer words. For example, the occupation ‘landscape gardener’ also contains the occupation ‘gardener’. We take as the occupation contained in the description field the occupation with the most number of words because the greater the number of words, the more specific the occupation. A description field may also contain more than one recognisable occupation. Through our own inspection, we note that the occupation listed first is most likely to represent the true occupation. Although at present we use only one occupation to represent the occupation in the description field, we have access to all the detected occupations. This will allow us to further investigate occupation in cases where the occupation is less clear cut.

### Handling Special Cases

Identifying an occupation is relatively straightforward compared to the processing required to handle the special cases that lead to false positives. False positives occur when our occupation detection tool outputs an occupation on the SOC2010 list that is not the occupation of the user, as determined by human validation. When we detect a false positive we add a rule to our occupation detection tool to improve it.

False positives occur for several reasons, the main source of which is the rarity of many of the occupations on the SOC2010 list. The SOC2010 list is an historic accumulation of all the occupations that have been recognised in the UK. As such, many of the occupations on the list are either rare or non-existent in modern times. For example, the occurrence of the word ‘devil’, which is an apprentice in a printing establishment that mixes tubs of ink and fetches type, is now more likely to refer to a sporting team, such as the Cardiff Devils ice hockey team.

Incorrect identification of occupation also occurs when recognised occupations occur as part of commonly occurring phrases. For example, the occupation ‘doctor’ appears as a word in the phrase, “Doctor Who fan”. In this case we must create a rule to handle ‘doctor’ because doctor is an occupation of modern times. Polysemy is also problematic when users describe themselves using a familial relationship, such as “father of two”. This is another source of misclassification because several familial relationships are also the names of occupations. For example, the relationships father and sister correspond to the positions of ‘Father’ and ‘Sister’ in the catholic church. Another noteworthy source of false positives is the historical link between surnames in the UK that derive from occupations such as Baker, Smith, Tanner and Turner. Although Baker is a surname that corresponds to an occupation in common use, surnames such as Smith, Tanner and Turner are no longer commonly held occupations. Because the input to our occupation detection tool is a piece of text, we can identify occupation from any source that provides a user-defined description field. An additional special exception was made for when a Tweeter could be identified as a ‘student’ as these users do not fall into an NS-SEC group (note that there were 4189 identified in the dataset).

### Results of Human Validation

The problems of automating occupation detection using Twitter data are complex and their interactions compound the difficulty in the development of explicit rules to identify occupations within the profile description field of Twitter users, hence the need for human intervention. Human validation is one way in which this type of human intelligence task (HIT) can be facilitated via an online community of ‘experts’ who can be tasked with verifying the accuracy of the occupation identification algorithm allowing us to test the hypothesis that the first recognisable SOC2010 occupation of a tweet is indeed the occupation of the Tweeter (as far as can be extrapolated from the limited profile description field). Online platforms such as CrowdFlower and Amazon Mechanical Turk provide an effective infrastructure and payment system for HITs such as image identification, online surveys, online experiments [35] and detecting tension in Twitter content [36] [17]. When human validation is used at this scale it is also known as crowdsourcing. For this study, the ‘experts’ are three of the authors with both substantive social science knowledge and co-designers of the rules from which we extract occupation using the COSMOS platform.

We randomly selected 1,000 cases out of the 32,032 to which an occupation was assigned and the three expert coders individually visually inspected the data, flagging cases where an occupation had been misidentified (type 1 errors). All three coders reviewed the same 1,000 cases and demonstrated a high level of inter-rater reliability (*Krippendorff’s Alpha = 0*.*73*). For 241 cases (24.1%) all three coders agreed that the occupation had been misclassified whilst 80 cases (8.0%) were flagged by two coders and 101 (10.1%) by a single coder, thus indicating a reasonable level of agreement on misclassifications between the coders for most of the type 1 errors. Table 2 lists the ten most frequent occupations for which a misclassification was flagged by all three coders. One reason for the high level of misclassifications around certain occupations is that Twitter profiles are used to express membership of particular interest groups (‘members’) or to advocate support for sports teams (‘supporter’). ‘Head’ was usually used as a prefix to a more specific occupation such as ‘head of services’ and thus denotes a position within the workplace rather than an occupation and ‘smith’ is an archaic occupational term that has been confused with the common name of ‘Smith’. Through post-data annotation discussions the three coders were in agreement that they had flagged ‘owner’ when used to refer to ownership of a pet, car or other material goods. ‘Former’ was often used as a prefix to an occupation from which someone has retired and ‘Professional’ was used in various instances to emphasise expertise in an occupation (e.g. ‘professional photographer’). ‘Master’ appeared often in a jovial sense such as ‘jack of all trades and master of none’, ‘general’ appeared in the context of ‘general manager’ and ‘runner’ referred to hobbies. This latter point demonstrates the importance of establishing a context in which to judge whether the occupation has been correctly identified as if an individual was indeed a runner in an occupational sense (a competitive athlete) then this would not be a misclassification, but for most of the instances that we encountered it was clearly a hobby. The same can be said for ‘drummer’, ‘singer’ and a whole host of other occupations.

**Table 2:** Ten most frequent misclassified occupations for three-coder agreement.

***Occupation***	***n***	***% of misclassified occupations***
*member*	*21*	*8.7*
*head*	*20*	*8.3*
*supporter*	*19*	*7.9*
*smith*	*10*	*4.1*
*owner*	*9*	*3.7*
*former*	*8*	*3.3*
*professional*	*8*	*3.3*
*master*	*7*	*2.9*
*runner*	*7*	*2.9*
*general*	*6*	*2.5*

An additional observation is that the level of three-way agreement differed substantially depending on NS-SEC group. 32.2% of assigned occupations had three-way agreement for NS-SEC 1 compared to 8.0% for NS-SEC 2, 26.5% for NS-SEC 3, 13.7% for NS-SEC 4, 7.1% for NS-SEC 5, 47.2% for NS-SEC 6 and 49.2% for NS-SEC 7. Clearly we can be more confident in some groups than others and this is a function of the types of occupations in said groups.

Following this, if we are to place faith in an automated occupation detection tool as applied to the whole dataset of 32,032 cases then we should reflect both what we could classify with reasonable certainty and what we cannot be confident in ascribing. The rules of classification can be adjusted incrementally to take into account the prefixes, such as ignoring ‘general’ when it precedes manager and likewise for suffixes when ‘owner’ follows words such as ‘car’ or ‘pet’. However differentiating the amateur hobbyist photographer from the professional is more challenging and our ability to do so relies on the tweeter providing a decipherable clue as to the distinction between hobby and occupation. Alternatively there are some occupations that could not be confused with hobbies that we can identify with confidence.

Table 3 provides examples of both the hobby/occupation problem and other jobs that are clearly demarcated from leisure activity for the 578 cases (57.8%) in which all three experts agreed that the correct occupation was identified. Occupations such as ‘teacher’, ‘manager’ and ‘councillor’ are not likely to be hobbies but there is an unusually high representation of creative occupations which could also be pursued as leisure interests with 4% of people in the dataset claiming to be an ‘actor’, 3.5% an ‘artist’ and 3.5% a ‘writer’. An alternative explanation is that Twitter is used by people who work in the creative industries as a promotional tool, meaning that the high proportion of users with these occupations is a function of how they use Twitter (i.e. to promote their skills, expertise or business). Either way we can only offer conjecture based on the limited information that we have, but it is clear that we can have greater confidence in ascribing occupation (and NS-SEC group) to individuals from particular sectors, most notably those in the professions which could not be confused with recreational interests.

**Table 3:** Occupations correctly identified (not flagged by any expert coder) where frequency = >5.

***Occupation***	***n***	***% of correctly classified occupations***	***Occupation***	***n***	***% of correctly classified occupations***
*actor*	*23*	*4.0*	*dancer*	*10*	*1.7*
*manager*	*22*	*3.8*	*singer*	*9*	*1.6*
*artist*	*20*	*3.5*	*chef*	*8*	*1.4*
*write*	*20*	*3.5*	*editor*	*8*	*1.4*
*teacher*	*17*	*2.9*	*coach*	*7*	*1.2*
*journalist*	*15*	*2.6*	*councillor*	*7*	*1.2*
*designer*	*14*	*2.4*	*personal trainer*	*7*	*1.2*
*photographer*	*12*	*2.1*	*model*	*6*	*1.0*
*director*	*11*	*1.9*	*producer*	*6*	*1.0*
*footballer*	*11*	*1.9*	*reporter*	*5*	*.9*
*owner*	*11*	*1.9*	*stylist*	*5*	*.9*

### Results of Class Breakdown

**Fig 1:**
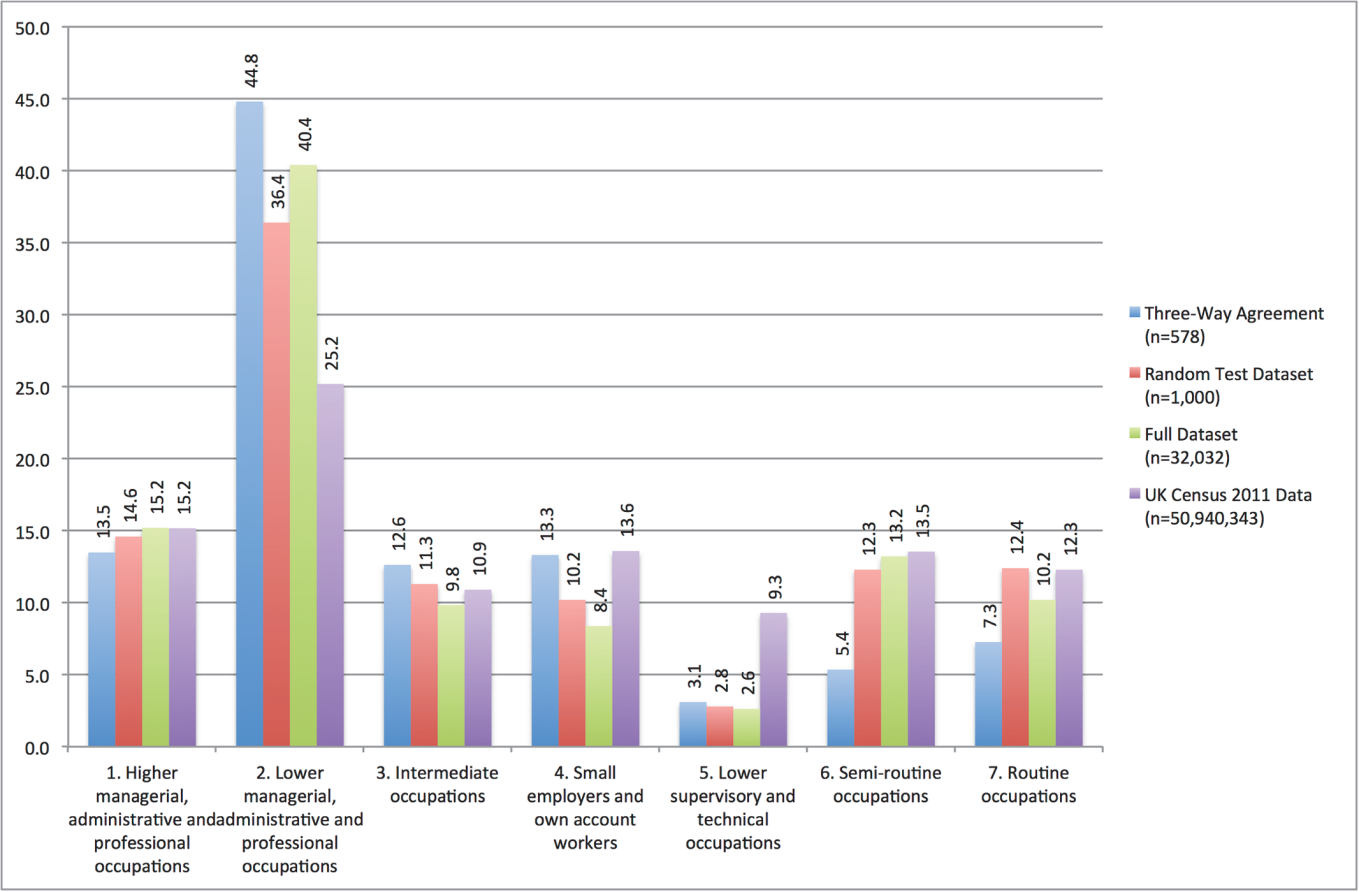
Proportion of individuals by NS-SEC group across four data sources

Having discussed the problems and uncertainties around identifying occupation, Fig. 1 presents a comparison NS-SEC group representation across four sources of data: the 578 cases in which all three expert coders agreed; the 1,000 cases randomly sampled for human validation; the full dataset of users and occupations totalling 32,032 cases (identified as unique through user ID); and the distribution of the UK population across NS-SEC groups according to the 2011 UK Census. The dataset containing the 32,032 identified occupations is available in the supporting information accompanying this paper (S1 Dataset). For the randomly sampled and full Twitter datasets we have not made any retrospective changes to the occupation detection rules to allow us to evaluate the difference between an *objective* naive rule set and *subjective* context based expert human judgements. We ascribe NS-SEC to SOC2010 codes using the look-up tables provided by the ONS [25] and we have collapsed ‘1.1: large employers and higher managerial and administrative occupations’ with ‘1.2: high professional occupations’ into a single category of ‘1: higher managerial, administrative and professional occupations’ on the basis that we do not have the data to estimate the size of the organisation that a tweeter works for. As there are no occupations for NS-SEC 8 (‘never worked and long-term unemployed’) we do not assign tweeters to this group.

The proportions of people in NS-SEC 1 (‘Higher managerial, administrative and professional occupations’) are similar across all four data sources indicating that the occupational detection tool is effective at classifying this group. The fact that there is little difference in proportions between cases where all three expert coders agreed and the random test dataset indicates that there are very few type one errors being made as instances of problematic occupational labels such as those illustrated in Table 2 are not frequent. This is an artefact of the unambiguous nature of occupational labels in NS-SEC 1 and the lack of frequency with which these job titles appear in everyday parlance. Words such as ‘doctor’, ‘solicitor’, ‘researcher’ and ‘engineer’ are clearly occupational terms which are both easy to detect through automation (without specifying exception rules) and provoke little to no disagreement between expert coders. When applied to the full dataset of tweeter profile descriptions containing occupation data the proportions shift only slightly, which is what we would expect as the sample is randomly drawn. A notable finding is that 15.2% of the UK population is located within NS-SEC 1 which corresponds exactly to the proportion of this group on Twitter, *ergo* the percentage representation of ‘higher managerial, administrative and professional occupations’ on Twitter is identical to that which we observe in the UK population (who may or may not partake in social media). This indicates that there is no bias for or against people in NS-SEC 1 engaging with Twitter (i.e. they are neither over nor under represented).

The level of consistency in proportions represented decreases substantially for NS-SEC 2. The gap of 8.4% points between cases with three-way agreement and the random test dataset is a function of misclassification in other NS-SEC groups, in particular groups 6 and 7 in which the expert coders disagreed with a substantial number of occupations identified. Regardless of the interplay and differences between the three Twitter-derived datasets, there is a clear overrepresentation of NS-SEC 2 occupations in the data compared with the general UK population which may be explained by the confusion between occupations and hobbies and/or the use of Twitter to promote oneself or one’s work. NS-SEC 2 is where occupations such as ‘artist’, ‘singer’, ‘coach’, ‘dancer’ and ‘actor’ are located and the utility of the tool for identifying occupation for this group is further exacerbated by the fact that this is by far the most populous group for Twitter users and the largest group in the general UK population by 10% points. Alternatively, if the occupation of these individuals has been correctly classified then we can observe that they are overrepresented on Twitter by a factor of two when using Census data as a baseline measure.

There is reasonable agreement between human coders and the automated tool for Intermediate occupations (NS-SEC 3), as demonstrated by the small difference in proportions between the three Twitter datasets. As with NS-SEC 1, the occupational titles in this group are largely unambiguous with jobs such as ‘teacher’, ‘nurse’, ‘soldier’ and ‘secretary’ although the most frequently occurring title in whole Twitter dataset is ‘writer’ (*n = 653*) which could be an occupation or hobby. NS-SEC 3 is present in the UK population at similar levels as represented on Twitter, thus indicating that our concern over misclassifying hobbies as occupations may be exaggerated for this group.

Following human validation the proportion of ‘small employers and own account workers’ (NS-SEC 4) increased in line with Census observations (this is where ‘photographer’ and ‘designer’ are located), but there is a clear under representation of ‘lower supervisory and technical occupations’ (NS-SEC 5) in the Twitter data. The consistency in proportion ascribed on the three-way agreement, random test and whole Twitter datasets indicates that the tool accurately identifies these occupations with respect to avoiding type 1 errors, meaning that either people from NS-SEC 5 are less likely to engage with Twitter than we might expect or that they do not publicise their occupations in the description of their profiles. One hypothesis for the over-representation of NS-SEC 2 on Twitter was that the social media platform is used to promote businesses and products by occupations that fall within this group, but NS-SEC 5 includes other occupations through which such publicity may increase business, particularly as it is dominated with trades (e.g. ‘electrician’, ‘plumber’, ‘mechanic’ and ‘locksmith’). It may be the case that Twitter is used to promote professional and creative industry services but not skilled trades, meaning that Twitter users from NS-SEC 5 may well be present and using Twitter but do not mention their occupation in their profile information.

Finally, for both NS-SEC 6 and 7 (‘semi routine’ and ‘routine’ respectively) the three-way agreement data significantly reduces the representation of these groups compared to the unmodified automated tool, although the latter does produce proportions aligned with the general UK population. A glance at the three most frequent occupations that fall into these groups (classified from the whole Twitter dataset) illustrates why so many type 1 errors occur. For NS-SEC 6 the top three occupations were ‘support’, ‘founder’ and ‘runner’ whilst for NS-SEC 7 they were ‘owner’, ‘page’ and ‘former’. These are obscure and uncommon occupations and the words themselves are more frequently used in common parlance with different meanings (see discussion around Table 2).

Interestingly, these findings differ to similar attempts at profiling using traditional survey methods. The Technology Tracker study conducted by Ipsos MORI (which uses a face-to-face survey methodology of around 1,000 participants) and found in Q3 2014 that 33% of UK Twitter users were from social grade AB [37]. Whilst NS-SEC and social grade are not necessarily synonymous, AB could be considered to contain similar occupations to NS-SEC 1 and 2 (55.7% of all Twitter users according to our study using the whole dataset). This may indicate an inflation of occupation by Twitter users and we discuss strategies for confirming/refuting this towards the end of the paper.

In summary, the unmodified occupation identification tool appears to be effective and accurate for NS-SEC groups in which occupational titles are unambiguous such as professions and skilled trades (NS-SEC 1, 3, 4 and 5). Where job titles are less clear or are synonymous with alternative activities (NS-SEC 2, 6 and 7) the requirement for human validation becomes apparent as the context of the occupational term must be taken into account such as the difference between “I’m a dancer in a ballet company” and “I’m a dancer trapped in the body of a software engineer”.

## Age

### Data

Schwartz et al. [10] demonstrate how to derive a Facebook user’s age based on the vocabulary used in their status updates. Their approach is innovative in that it does not simply prescribe *a priori* word or category lexicons associated with particular age groups, rather it takes the language used by the user (open-vocabulary analysis) and information about the age of the user in their online profile to identify words and phrases characteristic of particular age groups. Facebook is particularly suited to this type of analysis as the presence of demographic data provides a baseline from which proxies for demographic characteristics can be identified and tested. Similar work has been conducted on the blogosphere [38] which used factor analysis via a meaning extraction method to identify latent language concepts that explained the co-occurrence of particular words, such as a ‘poetic’ factor containing terms such as: eyes; heart, soul; pain; light; alone etc. Argamon et al. looked at the frequency of the occurrence of these factors and how they relate to both age groups and gender. Whilst this is a suitable approach for blogs in which lengthy pieces of prose can be dissected for analysis, it is not clear why such latent concepts will be observable over a series of micro-blogs where each tweet is an isolated piece of information.

Although Twitter profiles do not have an age field which can be extracted via the API, we can apply a similar approach to that of the occupational matching described above in which we identify the age of a user from their profile description. Consider another variant of the profile description referred to earlier:

*“Poker player*, *lecturer in social science and still 29 years young*!*”*

When an integer is followed by the word ‘year’ or a derivative such as ‘years’ we can be reasonably confident that the user is referring to their age. In addition, when an integer follows words and/or bigrams such as ‘age’ or ‘I am’ we can make a similar assumption. Alternatively a year of birth allows us to compute age and will generally be prefixed with ‘born’ or ‘born in’. For this latter example, we subtract the current year from the year of birth to calculate the age. Table 4 lists the conditions used to formulate the necessary rules to identify age data within the string field: Exceptions to these rules could relate to instances of people referring to time periods for which they’ve worked (“17 years working as a lecturer”) or refer to the age of another person (“I have a 4 year old daughter”). In light of this we specified the following pre and post integer rules in Table 5 to identify type 1 errors to ensure that the age detection tool can be generalised and automated for a very large number of cases with a minimum amount of error.

**Table 4:** Pattern Matching Rules for Identifying Age Data.

**Pre-Integer:**	**Post-Integer:**
*‘age’*	*‘years old’*
*‘aged’*	*‘yrs’*
*‘I’m’*	*‘yrs old’*
*‘I am’*	*‘years’*
*‘born’*	
*‘born in’*	

**Table 5:** Pattern Matching Rules for Identifying Type I errors.

***Pre-Integer*:**	***Post-Integer*:**
*‘for’*	*‘years as’*
*‘spent’*	*‘years working’*
	*‘years in’*
	*Any of the post-integer terms listed in Table 4 when followed by ‘son’, ‘daughter’*

Our pattern matching is limited by language and can only identify the age of users who have English language profiles so the actual number of identifications will be smaller, but as 40.35% of the content on Twitter is in the English language [20] we can be sure of identifying a substantial proportion of Twitter users. The sheer amount of data enables us to classify a large number of people and a small percentage of a large number is still large.

To develop our age detector we began by extracting text that contained patterns that look like they might be dates, such as text that contains the “DD/MM/YYYY” date format or age-related phrases such as “born in YYYY”. This gave us an early insight into the richness of age-related information that can be extracted from the description field of Twitter users. It also gave us an early insight into the difficulties. For example, Twitter users will happily provide detailed date of birth information in the form DD/MM/YYYY when they are describing their children or their pets. Adults, however, are more likely to provide their age in a phrase like “born in YYYY”.

Through a process of iterative testing and refinement, we settled on three comprehensive rules for age extraction using variations of the following phrases:

1. I am X years old
2. Born in X
3. X years old

where X can be a (typically) two-digit number or a date of the form DD/MM/YY or DD.MM.YYYY.

Each of these patterns must handle multiple and sometimes complex variations. To reduce the number of variations each pattern must match, we ignore case; that is we consider the phrase “Born in 1955” to be the same as “born in 1955”, even though the first phrase starts with a capital and the second phrase does not.

We apply our three age-extraction rules to each description field in the order presented above; the first rule to match an age wins. We found this particular order best handled the many cases in which one or more of the rules could return an age. For example, letting the “Born in X” rule win before the “X years old” rule means that the age of the following user is correctly calculated as 25 even though she hasn't updated her profile:

“*A 24 year old accountant born in 1989 with dreams of becoming an actress*.*”*

### Results of Age Identification

Unlike occupation derivation, identifying age is a simpler and less subjective exercise and a visual inspection of the 1,471 cases in which age could be identified using the ruleset above highlighted only one misclassification. In this single case a tweeter had written that their business was ‘born in 1887’ and was thus classified as being 127 years old. This is a difficult subtlety to avoid with automated techniques, but it is also an outlier and an incredibly rare turn of phrase as it was the only type 1 error out of a dataset of 32,032 cases and was deleted, leaving 1,470 cases. We can therefore be confident in the derivation of age from Twitter profile data, although the question of whether it is a true representation of the age of the tweeter must be considered (as discussed previously). The dataset containing the 1,470 identified ages is available in the supporting information accompanying this paper (S2 Dataset).

We might also hypothesis that young people are more likely to profess their age in their profile data and that this would lead to an overestimation of the ‘youthfulness’ of the UK Twitter population. As this is a new and developing field we have no evidence to support this claim, but the following discussion and estimations should be treated cautiously. What follows is our best attempt to profile the age distribution on Twitter in the absence of other baseline information (although we propose a research strategy for confirming demographic proxies towards the end of the paper).

It is worth drawing attention to the discrepancies between the age distribution as derived using our method and parallel observations made using terrestrial social research. Looking again at the results from the Technology Tracker study conducted by Ipsos MORI, nearly two thirds of Twitter users were under 35 years of age in Q3 of 2014 whereas our study clearly identifies 93.9% as being 35 or younger [37]. There are two possible reasons for this. The first is that the older population is less likely to state their age on Twitter. The second is that the age distribution in the survey data is a function of sample bias (i.e. participants over the age of 35 in the survey were particularly tech-savvy). This discrepancy between elicited (traditional) and naturally occurring (new) forms of social data warrants further investigation and we discuss how this could progress later in the paper.

**Fig 2:**
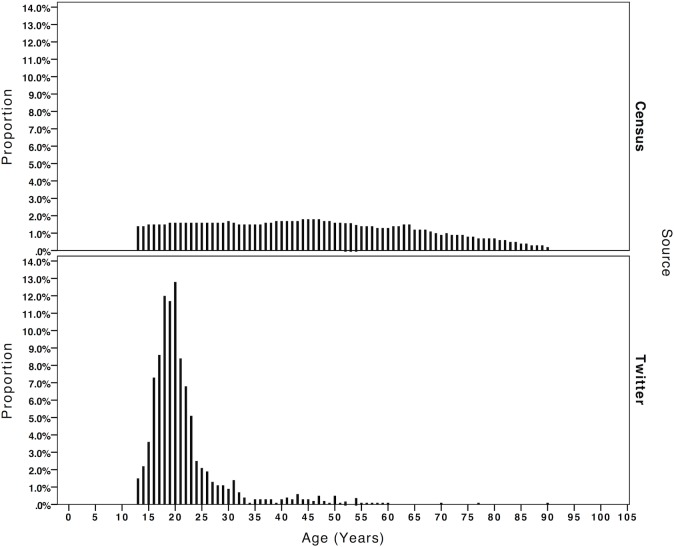
Comparison of age distribution between Twitter and the 2011 Census.

Assuming that the data is accurate and that there is no *a priori* reason to suspect that people lie about their age on Twitter (as opposed to dating sites, for example), Fig. 2 illustrates the significant difference in age distribution between the Twitter users and the UK population as of the 2011 Census. Parameters for the age range for both the Twitter and Census data were set at a minimum of 13 years and a maximum of 90 years because the terms and conditions of Twitter state that a user must be 13 years old or over and the oldest tweeter was aged 90. This means that direct comparison of the proportions on the *y* axis can be made. The population of Twitter users is much younger than the UK population with a peak around ages 16 to 22 accounting for 67.5% of all users, but there is also a long tail of older users with 5.0% of users of the age of 40 or above. Table 6 provides a breakdown of proportions by age groups and also an estimate of how many UK Twitter users fall into each category based on a UK Twitter user community of approximately 15 million [39].

**Table 6:** Breakdown of Twitter users by age groups.

***Age Group*:**	***Prop*. *of Users*:**	***Approx*. *Number of UK Users*:**
*13 to 20*	*59.4%*	*8*,*955*,*000*
*21 to 30*	*31.6%*	*4*,*680*,*000*
*31 to 40*	*4.4%*	*630*,*000*
*41 to 50*	*3.4%*	*510*,*000*
*51 to 60*	*1.1%*	*165*,*000*
*60+*	*0.3%*	*45*,*000*

The fact that younger people are more populous on social media as a proportion of the user population has been indicated by previous studies [40], but even the 1.1% of users between the ages of 51 and 60 could account for approximately 165,000 people in the UK. Taking this further and making the assumption that the age profile of users across the world is similar to the UK, we estimate the worldwide number of users between 51 and 60 years to be in the region of 2,981,000. Even this is a conservative estimate as it is calculated based 271,000,000 users who are active every month [41].

## Discussion

The methods proposed and tested for identifying the occupation and age of Twitter users have been presented in the same paper as the data required for their estimation originates from the same meta data field, however that is where the similarity ends. Matching key words from the description field to occupations shows variable success which appears to be related to NS-SEC groups through the definition of discrete professions and trades whilst identifying age is relatively simple with only one type 1 error identified in the whole dataset of 32,032 cases. However, of all the 398,452 unique profile descriptions we could only identify the ages of 1,470 tweeters (0.37%) whilst we found 32,032 profiles (8.10%) with an identifiable occupation. Even if we project the error rate of 42.2% onto all 32,032 profiles (where all three expert coders agreed) we still estimate to be able to identify a *meaningful* occupation for 18,638 UK Twitter users. To put it more succinctly, our age identification tool is more accurate and less prone to errors but it does not tell us about many people whilst our occupation identification tool tells us about a lot of people, but we have to temper our confidence in the measure.

One way to increase the application and utility of the age detection tool is to use the Twitter API to retrieve all the tweets ever produced by a user of whom we know the age and look at their use of words (frequency analysis), emoticons, syntax, hashtags and URLs to build predictive models similar to those run by Schwartz et al. [10] but tailored for Twitter micro-blogging. We hypothesise that a significant indicator of age would include the use of particular colloquialisms, nouns, adjectives and abbreviated phrases (e.g. ‘LOL’, ‘YOLO’ and ‘LMAO’) and therefore the presence of a given word increases or decreases the likelihood of belonging to a particular age group. This would be a reductionist approach that focuses on unigrams (single words) rather than the interactions between multiple words and as it is an open-vocabulary analysis we are not limited by a pre-existing lexicon of terms that is not reliant on *a priori* assumptions. The flexibility of this method means that we could model the predictive power of ‘emoticons’ and other non-alphanumeric phrases (such as: ‘:-D’; ‘:-$’; ‘;-p’ and so on) and we would expect these to feature significantly in enabling us to identify user age within a defined range.

Such an approach is suited for when the dependent variable is known and with age we now know that type 1 errors are rare to non-existent, although we can’t ignore the issue of whether tweeters are accurately reporting their age (ironically it may be that Twitter users who report their age are more accurate than Facebook users because the former *choose* to divulge the information publically whereas a user must customise their privacy settings on Facebook).Regardless, we have much less certainty when identifying occupation (as demonstrated with human validation) and there are so many occupations and such variability in jobs within NS-SEC groups that modelling language as a predictor of occupation and/or class is unlikely to be fruitful. Yet the comparisons between expert coders and the automated tool demonstrate that the accuracy of occupation identification and subsequent class is variable and that NS-SEC 1 and 3 are relatively easy to identify with precision (‘higher managerial, administrative and professional’ and ‘intermediate occupations’ respectively). We have already identified some of the confounders in occupation detection and future work should build upon this, incrementally adding new rules, testing their implementation and verifying their efficacy with human interpretation.

It is possible that Twitter data lends itself to other models of social class, most notably those focused around economic, social and cultural capital (recently advocated by Savage et al. [42]). In addition to the description field a researcher could look at archives of tweets for a user and list their hobbies, identify expenditure on luxury goods and leisure. A measure of network capital could even be constructed looking at number of followers and retweets (how influential a user is in a network) and whether an individual partakes in online communication interactions with other high capital users. Indeed, similar work has already been done around social capital in social networks such as Facebook [43]. These are all fruitful areas for future research.

### A Note on Ethics

While the derivation of social categories from social media data enhances their usefulness in social research, the ethics of using such data is still hotly debated. In our previous paper [20] we outlined the ethical issues of using social media data in social research. Since then, the content of a study conducted on Facebook was published that claims to have altered the emotions of users via tailored content that did not obtain informed consent from participants [44]. Facebook and the researchers received widespread criticism in the international media. Recent work by NatCen and COSMOS shows how users of social media platforms perceive their posts being used without their explicit consent [45][46]. The online pilot survey (n = 255) conducted by COSMOS into users’ perceptions of the use of their social media posts found that 73% knew that when accepting Terms of Service they were giving permission for some of their information to be accessed by third parties, and 82% were ‘not at all concerned’ or only ‘slightly concerned’ about university researchers using their social media information. The ESRC Framework for Research Ethics highlights the two key principles of informed consent and harm to participants. In relation to the former, it is not practically possible to seek informed consent from Twitter users in big ‘social’ data research. Twitter’s Terms of Service require users to provide their consent for Twitter to share any content posted with third parties. We may therefore argue that researchers in this field must accept that consent has been provided. But we must interpret these Terms of Service within the framework of our social science informed ethical and moral architecture [47]. As such, we suggest presenting results at an abstract and aggregate level. Statistical analyses of online social networks afford this level of abstraction, as no direct quotations of users are presented in published work. Presenting results in this way obscures all personal information, maintaining anonymity and minimising harm (for a fuller discussion see Williams et al. [46]). While these techniques may provide some comfort to researchers and participants in the short-term, we acknowledge the need for further work to be done in this area to assure maximum protection for all concerned.

## Conclusions

In this paper we have specified, implemented and critically evaluated two new tools for extracting demographic data from Twitter user profiles—age and occupation (leading to class). We found that we are able to identify certain occupational groups with more accuracy and less error than others, most notably those with job titles which are still in common parlance and are not used frequently in other contexts (such as ‘runner’). The over-representation of Twitter users in NS-SEC 2 is either due to the misclassification of hobbies as occupations or a disproportionately large number of people from the creative industries engaging with Twitter. The next step is to validate this tool through establishing the ground-truth via ascertaining the occupation of tweeters through alternative means, such as social surveys (an on-going programme of work for the authors).

Social desirability effects aside, we demonstrated next to no type 1 errors for our age detection tool through following a relatively short set of pattern matching rules. We found that the age distribution of Twitter users is much younger than the UK population as of the 2011 Census, with 67.5% of all users between 16 and 22 years. Despite the under-representation of older tweeters, our projections indicate that there may be over half a million Twitter users over the age of 40 in the UK. Using techniques adapted from previous researchers we can look at the content of these tweets to build predictive models of age, enabling us to classify a larger proportion of UK users—although confirmation of the accuracy of age information is also important and can also be done through cross referencing with social survey data.

These findings illustrate an incremental step forwards on the repurposing of social media data for social scientific analysis. Establishing demographic data for Twitter users is a key challenge of 21st Century social science and we encourage the research community to try and test the methods that we have proposed in the hope that this contribution will point others in a fruitful direction.

## Supporting Information

S1 Dataset Identified occupations of tweeters with SOC2010 and NSSEC groups listed. (CSV)

S2 Dataset Identified ages of tweeters. (CSV)
